# Daytime nap and nighttime breastfeeding are associated with toddlers’ nighttime sleep

**DOI:** 10.1038/s41598-021-81970-6

**Published:** 2021-02-04

**Authors:** Machiko Nakagawa, Hidenobu Ohta, Rinshu Shimabukuro, Yoko Asaka, Takayo Nakazawa, Yoshihisa Oishi, Michio Hirata, Akiko Ando, Takashi Ikeda, Yuko Yoshimura, Yusuke Mitani, Yousuke Kaneshi, Keita Morioka, Rika Fukutomi, Kyoko Kobayashi, Miwa Ozawa, Masahiro Takeshima, Kazuo Mishima, Mitsuru Kikuchi, Kazutoshi Cho, Hitoshi Yoda, Isao Kusakawa

**Affiliations:** 1 Department of Pediatrics, St. Luke’s International Hospital, 9-1 Akashi-cho, Chuo-ku, Tokyo 104-8560 Japan; 2 Pediatric Nursing, Graduate School of Nursing Science, St. Luke’s International University, 10-1 Akashi-cho, Chuo-ku, Tokyo 104-0044 Japan; 3 Department of Neonatology, Toho University Omori Medical Center, 6-11-1 Omori-nishi, Ota-ku, Tokyo 143-8541 Japan; 4 Department of Neuropsychiatry, Akita University Graduate School of Medicine, Hondo 1-1-1, Akita, Akita 010-8543 Japan; 5 Department of Sleep-Wake Disorders, National Institute of Mental Health, National Center of Neurology and Psychiatry, 4-1-1 Ogawa-higashi-cho, Kodaira, Tokyo 187-8553 Japan; 6 Department of Psychiatry, Asai Hospital, 38-1 Togane, Chiba, 283-0062 Japan; 7 Faculty of Health Sciences, Hokkaido University, N12, W5, Kita-ku, Sapporo, 060-0812 Japan; 8 Maternity and Perinatal Care Center, Hokkaido University Hospital, N15, W7, Kita-ku, Sapporo, 060-8638 Japan; 9 Department of Pediatrics, Japanese Red Cross Medical Center, 4-1-22 Hiroo, Shibuya-ku, Tokyo 150-8935 Japan; 10 Research Center for Child Mental Development, Kanazawa University, 13-1 Takara-machi, Kanazawa, 920-8640 Japan; 11 Institute of Human and Social Sciences, Kanazawa University, Kakuma-machi, Kanazawa, 921-1192 Japan; 12 Department of Pediatrics, Kanazawa University, 13-1 Takara-machi, Kanazawa, 920-8640 Japan

**Keywords:** Health care, Medical research

## Abstract

The purpose of the present study is to examine the association between toddlers' sleep arrangements and their nighttime sleep duration and other sleep variables. For this investigation, we performed a study in which child activity and sleep levels were recorded using actigraphy. The parents of 1.5-year-old toddlers (n = 106) were asked to attach an actigraphy unit to their child’s waist with an adjustable elastic belt and complete a sleep diary for 7 consecutive days. Questionnaires were used to assess the sleep arrangements of the toddlers. There was a significant negative correlation between nap duration and nighttime sleep duration, suggesting that longer nap sleep induces shorter nighttime sleep duration. Among the sleep arrangements, such as nighttime breastfeeding or co-sleeping, only nighttime breastfeeding predicted shorter nighttime sleep duration. Our findings indicate that shorter naps induce a longer nighttime sleep in 1.5-year-old toddlers while nighttime breastfeeding decreases their nighttime sleep duration.

## Introduction

Young children experience marked changes in the amount and distribution of nap and nighttime sleep during the first five years of life, in which the frequency of daytime naps decreases and the biphasic sleep–wake pattern gradually disappears to move to a consolidated nighttime sleep like in adults^1–5^. Sleep problems are commonly found in toddlers, with prevalence estimates of 25% among children across the world^6,7^. Previous research indicates that sleep arrangements are a strong predictive factor for toddler's sleep problems. For instance, co-sleeping was demonstrated to be associated with persistent child night wakings and bedtime struggles^2,8–10^ and also infants' poor sleep quality^10–12^, according to reports by mothers. In another recent study, falling asleep independently was associated with longer nighttime sleep duration and fewer night wakings, whereas other sleep arrangements, such as co-sleeping or room-sharing, were not^13^. Another previous study on mother-infant dyads with 3 to 18-month-old children demonstrated that, in comparison to solitary sleep, room-sharing is associated with more objective sleep disturbances in mothers but hardly any in infants^13,14^. In addition, breastfeeding has been found to be associated with more nocturnal wakings^15^, but has not been found to be linked to shorter nighttime sleep duration in children^13^. However, despite the extensive number of studies performed to date, the best sleep arrangements for toddlers is still a much debated issue^16^.

In a recent study, we investigated the sleep properties of young toddlers approximately 1.5 years of age and reported that nap duration controls the distribution ratio between nap and nighttime sleep^17^. In the same study, however, we did not examine whether sleep arrangements such as co-sleeping or nighttime breastfeeding influence nighttime sleep. In the current study, to gain an answer to this question, we examined the relationship between the sleep properties and sleep arrangements of 106 toddlers who had been born mature (full term toddlers). Unlike the previous report^14^, our current study includes data on co-sleeping with parents, which has been less reported in western sleep culture, where bed-sharing is not as common as in Asian countries^18^. Questionnaires to parents were used to assess the sleep arrangements of the toddlers. This is the first actigraphic study to examine the effects of co-sleeping and/or nighttime breastfeeding on the nighttime sleep of toddlers approximately 1.5 years of age—an age at which a regular child health examination is performed nationwide in Japan.

## Results

### Sleep properties of the toddlers

**Figure 1:**
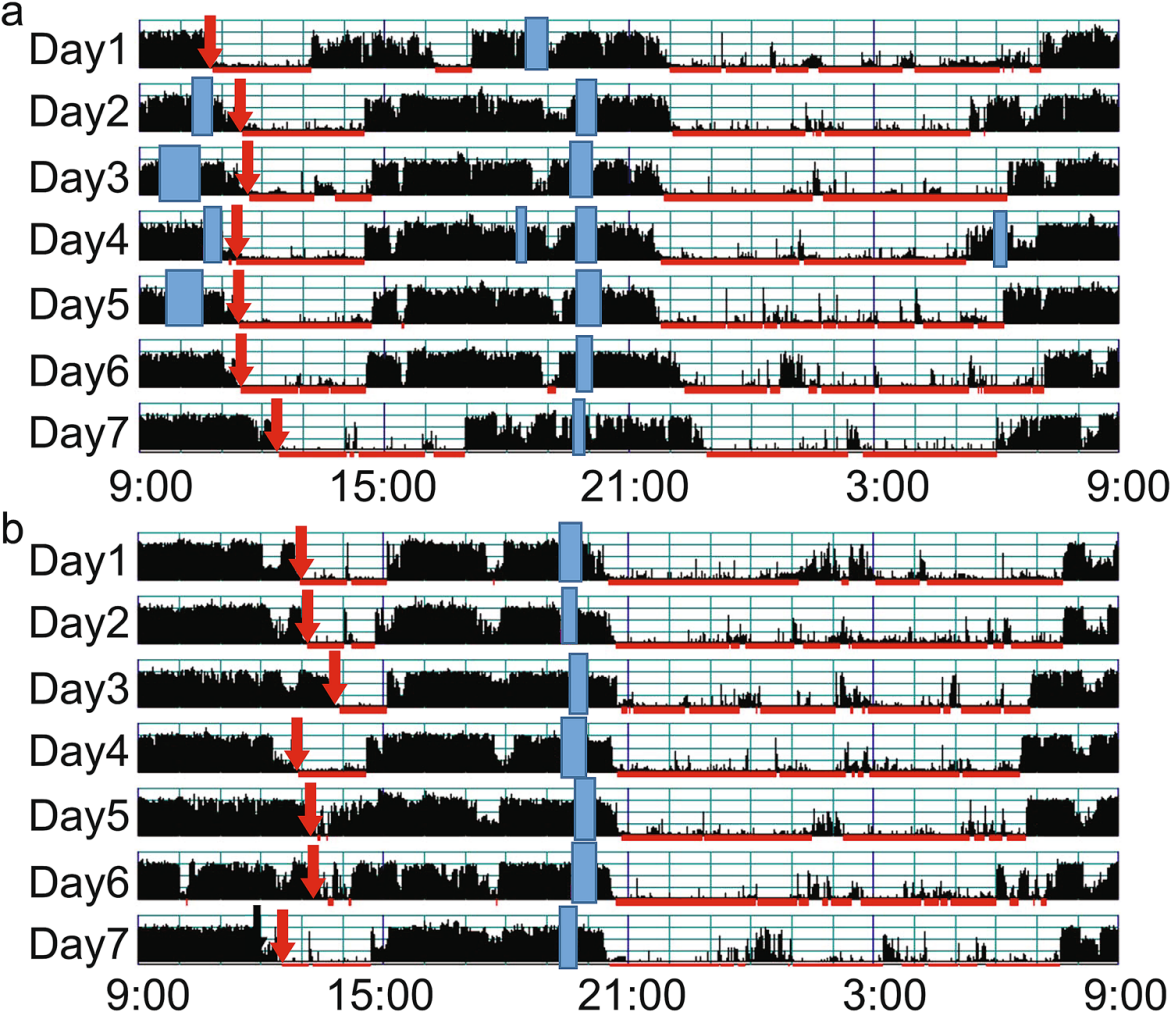
The actograms show representative daily activity-rest patterns of toddlers of approximately 1.5 years of age with early nap onset times and long nap duration (**a**) and late nap onset times and short nap duration (**b**). The vertical axis shows the 7 consecutive observation days and the horizontal axis shows the course of each 24 h day from 9:00 h (9:00 am). Activity counts per minute are represented by the height of the vertical black bars on each actogram. The red arrows and the blue rectangles indicate naps and bathing periods, respectively. The red underlines are the periods that were automatically judged as sleep periods by the actigraph software. Note that the nap onset times are recognized as relatively early in (**a**) but as late in (**b**).

**Figure 2:**
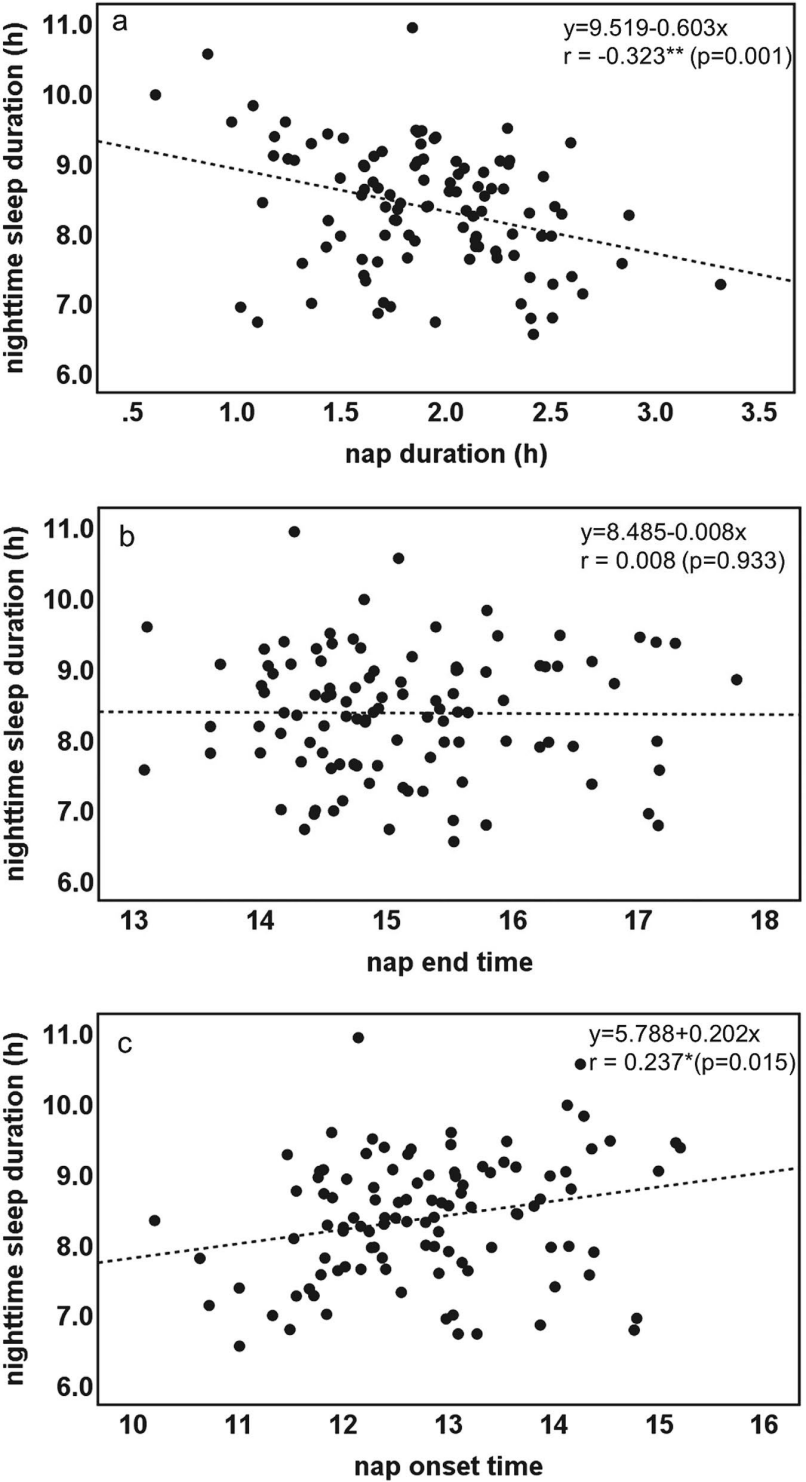
Correlations of nighttime sleep duration with nap duration (**a**), nap end time (**b**), and nap onset time (**c**) in term toddlers of approximately 1.5 years of age (**p < 0.01, *p < 0.05).

**Figure 3:**
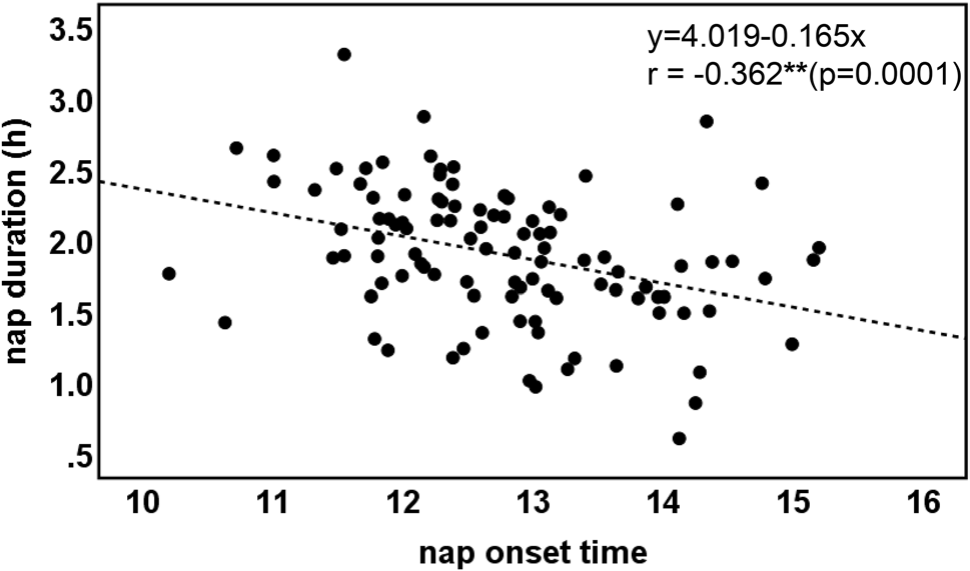
Correlations of nap duration with nap onset time in term toddlers of approximately 1.5 years of age (**p < 0.01).

The characteristics of the 106 toddlers are demonstrated in Table 1. The toddlers’ sleep arrangements are shown in Table 2. The toddlers’ sleep variables such as bedtime, wake time, nighttime sleep duration, and nap duration are shown in Table 3. No differences were found between boys and girls in any of the sleep variables (t-test, *p* > 0.05). Figure 1 shows the representative daily activity-rest patterns of the approximately 1.5-year-old toddlers, indicating the existence of various nap patterns among the toddlers. A significant negative correlation between nap duration and nighttime sleep duration was found (r = − 0.323, *p* = 0.001), suggesting that longer nap duration induces shorter nighttime sleep duration (Fig. 2a), as we previously reported^17^. Also, unlike our previous report, no significant correlation was observed between nap end time and nighttime sleep duration (Fig. 2b). In addition, we found a significant correlation between nap onset time and nighttime sleep duration (r = 0.237, *p* = 0.015), which indicates that earlier nap onset time leads to shorter nighttime sleep duration (Fig. 2c). This correlation between nap onset time and nighttime sleep duration may be affected by the negative correlation between nap onset time and nap duration (r = − 0.362, *p* = 0.0001), suggesting that earlier nap onset induces longer nap duration which in turn leads to shorter nighttime sleep duration (Fig. 3). To confirm this assumption, we also performed mediation analysis to examine the relationship between nap onset time and nighttime sleep duration mediated by nap duration. As Supplementary Figure 1 illustrates, the standardized regression coefficients between nap onset time and nap duration (a =  −0.362, *p* < 0.001), nap duration and nighttime sleep duration (b =  −0.237, *p* = 0.007), and nap onset time and nighttime sleep duration (c = 0.237, *p* = 0.015) were statistically significant. Mediation analysis also demonstrated that the indirect effect of the relationship between nap onset time and nighttime sleep duration via nap duration was significant (a x b = 0.099, *p* = 0.023). However, the direct effect in the absence of the mediator was not significant (c’ = 0.138, *p* = 0.192). The results showed that shorter nighttime sleep duration in infants with earlier nap onset time was induced by longer nap duration.

**Table 1:** Characteristics of participants.

Gestational age at birth (weeks), mean ± s.d	39.6 ± 1.2
Birth weight (g), mean ± s.d	3073 ± 385
No. of toddlers (boys:girls)	106 (50:56)
Maternal age at birth, mean ± s.d	35.6 ± 4.4
*Birth order*	
First born	72
Subsequent born	34
Months of age at actigraphic recording, mean ± s.d	19.2 ± 0.9

**Table 2:** Sleep arrangements and sleep variables by gender (number or mean ± s.d.).

		Boys (n = 50)	Girls (n = 56)	*p* value
*Home environment*				
Siblings	Yes	18	16	
	No	32	40	0.418
Child having own room	Yes	4	2	
	No	46	54	0.329
Co-sleeping with parents	Yes	39	39	
	No	11	17	0.335
Nighttime breast feeding				
Yes		12	19	
No		38	37	0.266
*Putting children to sleep with formula*				
Yes		3	6	
No		47	50	0.390
*Nap during weeks*				
Yes		50	56	
No		0	0	
*Bed time*				
Weekday	21:06 ± 0:47	21:01 ± 0:46	21:11 ± 0:48	0.277
Weekend	21:10 ± 0:52	21:09 ± 0:47	21:11 ± 0:56	0.856
*p* value	0.544	0.371	0.976	
*Wake time*				
Weekday	7:03 ± 0:48	6:57 ± 0:46	7:09 ± 0:48	0.227
Weekend	7:09 ± 0:53	7:03 ± 0:53	7:15 ± 0:52	0.229
*p* value	0.403	0.614	0.508

**Table 3:** Sleep variables (mean ± s.d.).

Sleep variables	Total (n = 106)	Boys (n = 50)	Girls (n = 56)	*p* value
Bedtime	21:07 ± 0:46	21:04 ± 0:44	21:10 ± 0:48	0.444
Sleep onset time	21:35 ± 0:47	21:33 ± 0:46	21:37 ± 0:48	0.639
Wake time	7:04 ± 0:46	6:59 ± 0:45	7:10 ± 0:46	0.217
Sleep latency	27.7 ± 12.0	28.6 ± 12.5	27.0 ± 11.6	0.494
Nighttime sleep duration	8.37 ± 0.88	8.32 ± 0.79	8.42 ± 0.96	0.576
Nap duration	1.90 ± 0.47	1.94 ± 0.42	1.87 ± 0.51	0.500
Total sleep duration	10.26 ± 0.86	10.24 ± 0.80	10.28 ± 0.91	0.826
Nap onset time	12:46 ± 1:01	12:50 ± 1:04	12:43 ± 0:59	0.534
Nap end time	15:07 ± 0:57	15:11 ± 0:57	15:04 ± 0:58	0.499
Sleep efficiency	88.7 ± 7.0	88.6 ± 6.9	88.8 ± 7.2	0.881
WASO (wake after sleep onset)	63.6 ± 39.8	63.9 ± 39.8	63.2 ± 40.2	0.930

### Effects of sleep variables and sleep arrangements on toddlers’ nighttime sleep duration

Before logistic analysis, univariate analysis was performed in order to select possible variables associated with nighttime sleep duration (see Table 4). Next, to evaluate factors contributing to the toddlers’ nighttime sleep duration, we performed a logistic analysis for the effects of gender, perinatal conditions, nap variables, nighttime sleep variables, and sleep arrangements on the nighttime sleep duration of the toddlers (Table 5). According to analysis of nighttime sleep duration in model 1, which was adjusted for birth profile characteristics such as birth order, birth weight, and maternal age, no significant odds ratios (ORs) for toddlers with a nighttime sleep duration of ≥ 8.37 h (mean) were found. In model 2, which adds the sleep variables of nap duration, nap onset time, and wake time to model 1, the ORs for toddlers with a nighttime sleep duration of ≥ 8.37 h (mean) were 0.260 (*p* = 0.005) for nap duration, indicating that longer nap duration is a significant predictor of shorter nighttime sleep duration in toddlers, but failed to find any significant correlations with nap onset time or wake time. In model 3, which adds the sleep arrangement factors of nighttime breastfeeding, “child having own room”, and putting children to sleep with formula to model 2, the ORs for nighttime sleep duration of ≥ 8.37 h (mean) were 0.233 (*p* = 0.011) for nap duration, 1.717 (*p* = 0.038) for nap onset time, and 0.110 (*p* = 0.0001) for nighttime breastfeeding, indicating that longer nap duration, earlier nap onset time, and nighttime breastfeeding are significant predictors of shorter nighttime sleep duration in toddlers. However, there were no significant correlations between nighttime sleep duration and other sleep arrangement factors.

**Table 4:** The associations of gender/birth profiles, sleep variables, and sleep arrangement factors and nighttime sleep duration evaluated by univariate analysis (**p < 0.01, *p < 0.05).

Sleep-related factors	r	R^2^	*p* value
**Gender and birth profiles**			
Maternal age at birth	− 0.202*	0.041	0.038
Birth order	− 0.184	0.034	0.059
Birth weight	− 0.128	0.016	0.190
Gender	− 0.055	0.003	0.576
**Sleep variables**			
Total sleep duration	0.847**	0.718	0.000
Sleep efficiency	0.794**	0.630	0.000
WASO	− 0.739**	0.546	0.000
Night wakings	− 0.638**	0.408	0.000
Nap duration	− 0.323**	0.104	0.001
Wake time	0.247*	0.061	0.011
Nap onset time	0.237*	0.056	0.015
Sleep onset time	− 0.231*	0.053	0.017
Bed time	− 0.220*	0.048	0.024
Daily variation in sleep onset time	− 0.086	0.007	0.380
Sleep latency	− 0.041	0.002	0.679
Nap end time	− 0.008	0.000	0.933
Daily variation in wake time	− 0.003	0.000	0.979
**Sleep arrangement factors**			
Nighttime breast feeding	− 0.314**	0.098	0.001
Child having own room	− 0.156	0.024	0.111
Putting children to sleep with formula	0.139	0.019	0.154
Co-sleeping with parents	− 0.076	0.006	0.442

**Table 5:** Logistic regression of nighttime sleep of toddlers with gender/birth profiles, sleep variables, and sleep arrangement factors (OR, 95% CI **p < 0.01, *p < 0.05).

Variables	Model 1,OR (C.I.)	Model 2,OR (C.I.)	Model 3, OR(C.I.)
Maternal age	N.S	N.S	N.S
Birth order	N.S	N.S	N.S
Birth weight	N.S	N.S	N.S
Nap duration		0.260 (0.103, 0.660)**	0.233 (0.076, 0.718)*
Nap onset time		N.S	1.717 (1.031, 2.860)*
Wake time		N.S	N.S
Nighttime breast feeding			0.110 (0.036, 0.343)**
Child having own room			N.S
Putting children to sleep with formula			N.S
*p* value	N.S	0.002	0.000
R^2^ (Cox–Snell)	N.S	0.083	0.266

## Discussion

The present study makes two significant findings concerning the sleep properties of toddlers at approximately 1.5 years of age. First, the multivariate analysis (Table 5) indicates that nighttime breastfeeding, rather than sleep arrangements, such as co-sleeping, is significantly associated with the nighttime sleep duration of toddlers. This is also in line with a previous actigraphy study by Yoshida et al.^19^, which found bed-sharing to be associated only with night wakings and not with nighttime sleep duration. However, previous questionnaire studies by Lo et al.^20^ reported that co-sleeping was associated with shorter nighttime sleep duration. In addition, questionnaire studies by Mindell et al.^21^ and Yu et al.^13^ reported that breastfeeding was not associated with nighttime sleep duration. The reason for the discrepancy between these reports and ours could be because our study employed actigraphy for sleep assessment while the conflicting previous studies did not. Actigraphy is generally regarded as superior to maternal reports in estimating child sleep–wake activity^22,23^, because some mothers tend to underestimate child waking frequencies and durations^1^ while others tend to overestimate them^14,22^.

The second significant finding, also from the multivariate analysis (Table 5) and mediation analysis (Supplementary Figure 1), was a possibility that longer nap duration significantly shortens the nighttime sleep duration of toddlers. This finding is consistent with the results from other studies using questionnaires or actigrapy^1,5,24–26^ and also from our own previous actigraphic study^17^, which all reported that longer nap duration induces shorter nighttime sleep duration. Although the present study is an association study that shows correlation between nighttime sleep duration and the other sleep variables and sleep arrangement factors, we performed mediation analysis to assess the effect of nap duration on the relationship between nap onset time and nighttime sleep duration. As a result, the analysis indicated that the effect of nap onset time on nighttime sleep duration was significantly mediated by nap duration. Therefore, it may be advantageous for caregivers to avoid setting early nap onset time and late nap end time to reduce the length of toddlers' nap duration, which would result in longer nighttime sleep durations. This result differs from what the results of our previous study suggested, that only a later nap end time induces a shorter nighttime sleep^17^. This discrepancy in results could be due to the difference in statistical analysis between the two studies. Unlike our current study, our previous study^17^ did not perform a multivariate analysis which included both sleep variables and sleep arrangement factors.

There are three matters concerning the current study that warrant consideration. First, although the sleep habits of toddlers are affected by those of their parents, especially mothers^27^, the present study did not investigate the sleep habits of the parent themselves. Secondly, the sleep habits of toddlers are also affected by socio-cultural environments, socioeconomics, individual family ethics, income and the educational backgrounds of parents^28^. The details of these were not obtained in the present study. Thirdly, the present study was an association study and was not able to fully investigate the effect of nap duration because we did not perform an RCT study, which would require exposing children to at least two different nap durations. Such an RCT study would further strengthen the findings of the present study that indicate that daily naps with short periods would contribute to longer nighttime sleep in toddlers of approximately 1.5 years of age.

During their early developmental stages, young toddlers have bi-phasic sleep patterns which include both napping and nighttime sleep. There is still an ongoing debate whether either nap or nighttime sleep is more valuable to achieve proper physical and cognitive development or if only total sleep duration of nap and nighttime sleep is an important factor for healthy child development (total sleep duration itself in this study was significantly associated with sleep arrangement factors such as nighttime breastfeeding and child having own room as shown in Supplementary Table 1 and 2). Several clinical studies have confirmed positive associations between nighttime sleep duration and toddlers’ physical growth and cognitive development, while other studies have not^29,30^. However, some recent studies have at least reported that daytime naps improve word learning in toddlers of 12, 15 and 16 months of age^31–33^. Previous clinical studies have also demonstrated a positive association between nighttime sleep duration and the cognitive development of toddlers of 10, 11, and 13 months of age^34,35^. A further study using additional physiological and psychological parameters will be required to obtain appropriate answers regarding sleep issues in child development.

In summary, our findings suggest that duration-controlled naps and the cessation of nighttime breastfeeding can induce a longer nighttime sleep duration in toddlers without the need to stop co-sleeping. Unlike adult’s nighttime sleep, toddlers’ nighttime sleep is significantly affected by their unique developmental sleep-related factors, i.e., daytime naps and nighttime breastfeeding.

## Methods

### Participants

Young toddlers of approximately 1.5 years of age were recruited at the Children’s Clinic of St. Luke’s International Hospital (Tokyo, Japan). Inclusion criteria were as follows: (1) term pregnancy (defined as being born at at least 37 weeks’ gestational age) and (2) the absence of chromosomal or other major genetic abnormalities, suspected neuromuscular disorders or significant chronic lung disease. Exclusion criteria was parental language difficulties. Of 129 eligible toddlers, 23 were excluded because sleep data were invalid due to technical problems with the activity recording devices or incomplete description of sleep diary. The final sample thus consisted of 106 young toddlers (50 boys, 56 girls). The ethics committee of St. Luke’s International Hospital approved the study protocol (UMIN000021153) and all procedures were carried out in accordance with the approved guidelines. Written informed consent was obtained from the parents.

### Activity and sleep assessment

#### Actigraphy

For activity and sleep measurement we used actigraphy, as previously described^17^. Actigraphy employs a miniature wristwatch-like accelerometer which is attached to the wrist, ankle or waist and continuously records movement for an extended period. The actigraphy device used in the present study was the Actigraph (Micro-mini RC, Ambulatory Monitoring Inc., NY, USA). The parents were asked to attach an Actigraph to their child’s waist with an adjustable elastic belt for 7 consecutive days. Waist attachment was chosen as we found it less disturbing than wrist or ankle attachment. Previous studies have also demonstrated that a minimum of 7 nights was necessary to obtain reliable data^36^. The actigraphs were removed from the toddlers only for bathing—the average time of which was 29.7 ± 14.2 min.

Motility levels were sampled in the zero-crossing mode in 1-min epochs. The resolution of the Actigraphs was set at 0.01 G/s. The activity data recorded by the Actigraphs was later downloaded using ActMe software (ver. 3.10.0.3, Ambulatory Monitoring Inc.), and then sleep measurements were analyzed with Sadeh’s algorithm^37,38^, using Action-W software (ver. 2.4.20, Ambulatory Monitoring Inc.).

### Sleep diary and questionnaire

Parents were instructed to complete a sleep diary for the 7-day period while their child was wearing the Actigraph. The diary consisted of seven 24-h single-sheet schedules, on which parents were asked to write information such as time of nap, going in/out of bed, bathing and night wakings of which they were aware. At the same time, questionnaires (Supplementary Data 2) given to parents were used to assess the daily sleep arrangements of the toddlers during the same 7-day study period.

### The computation of nighttime sleep and nap

The longest sleep period of the day starting after 19:00 was defined as “nighttime sleep”, and all sleep periods of 11 min or more starting before 19:00 were defined as “nap”. Daily nap duration was calculated as the average total nap time per day.

### Statistical analysis

Univariate analysis was performed before logistic regression analysis (see Table 4). The degrees of correlation between nighttime sleep duration and gender/birth profiles, sleep variables, and sleep arrangement factors were assessed using the Spearman correlation test. Only variables with relatively significant values (*p* < 0.2) in the Spearman correlation tests were included in logistic regression analysis. Logistic regression was used to calculate odds ratios (OR) with 95% confidence intervals as estimates of effects, with nighttime sleep duration of toddlers as the outcome variable.

We used 3 models to investigate the effects of gender/birth profiles, sleep variables, and sleep arrangement factors on nighttime sleep duration (see Table 5). Model 1 included gender and perinatal conditions. In model 2, we added the nap variables of nap duration, nap onset time and wake time to model 1. In model 3, we added the sleep arrangement factors of nighttime breastfeeding, putting children to sleep with formula, and “child having own room” to model 2.

Univariate analysis and logistic regression analysis were performed with SPSS Statistics 25.0 (IBM Corp. Armonk, NY, USA). Summary measurements are presented as means ± s.d.s. Spearman correlation was used to assess associations between variables. The gender difference in sleep arrangements and bedtime routine was analyzed using a χ^2^ test for categorical data and a t-test for continuous data.

We also explored the possible mediating role of nap duration in the relationship between nap onset time and nighttime sleep duration as the outcome. Mediation analysis was conducted using R software 4.0.2 (R Foundation for Statistical Computing, Vienna, Austria, https://www.R-project.org/) with mediation package^39^. The significance of the direct and indirect (mediation) effects with 95% CI was tested using a non-parametric bootstrapping procedure (10,000 bootstrap samples).

## Supplementary Information

Supplementary Information.
