# Supplementary material for: Daytime nap and nighttime breastfeeding are associated with toddlers’ nighttime sleep
Source: Sci Rep. 2021 Feb 4;11:3028. doi: 10.1038/s41598-021-81970-6 (PMC7862350; doi:10.1038/s41598-021-81970-6)
Supplement: Supplementary file 1 — Supplementary Information. [file 41598_2021_81970_MOESM1_ESM.docx]

**Supplementary Information**

Daytime nap and nighttime breastfeeding are associated with toddlers’ nighttime sleep

**Authors:**

Machiko Nakagawa^1,2,3^, Hidenobu Ohta^4,5,6^*, Rinshu Shimabukuro^1^, Yoko Asaka^7^, Takayo Nakazawa^8^, Yoshihisa Oishi^9^, Michio Hirata^1^, Akiko Ando^8^, Takashi Ikeda^10^, Yuko Yoshimura^10,11^, Yusuke Mitani^12^, Yousuke Kaneshi^8^, Keita Morioka^8^, Rika Fukutomi^2^, Kyoko Kobayashi^2^, Miwa Ozawa^1^, Masahiro Takeshima^4^, Kazuo Mishima^4^, Mitsuru Kikuchi^10^, Kazutoshi Cho^8^, Hitoshi Yoda^3^, Isao Kusakawa^1,2^

^1^ Department of Pediatrics, St. Luke’s International Hospital, 9-1 Akashi-cho, Chuo-ku, Tokyo 104-8560, Japan

^2^ Pediatric Nursing, Graduate School of Nursing Science, St. Luke's International University, 10-1 Akashi-cho, Chuo-ku, Tokyo 104-0044, Japan

^3^ Department of Neonatology, Toho University Omori Medical Center, 6-11-1 Omori-nishi, Ota-ku, Tokyo 143-8541, Japan

^4^ Department of Neuropsychiatry, Akita University Graduate School of Medicine, Hondo 1-1-1, Akita, Akita 010-8543, Japan

^5^ Department of Sleep-Wake Disorders, National Institute of Mental Health, National Center of Neurology and Psychiatry, 4-1-1 Ogawa-higashi-cho, Kodaira, Tokyo 187-8553, Japan

^6^ Department of Psychiatry, Asai Hospital, 38-1 Togane, Chiba 283-0062, Japan

^7^ Faculty of Health Sciences, Hokkaido University, N12, W5, Kita-ku, Sapporo 060-0812, Japan

^8^ Maternity and Perinatal Care Center, Hokkaido University Hospital, N15, W7, Kita-ku, Sapporo 060-8638, Japan

^9^ Department of Pediatrics, Japanese Red Cross Medical Center, 4-1-22 Hiroo, Shibuya-ku, Tokyo 150-8935, Japan

^10^ Research Center for Child Mental Development, Kanazawa University, 13-1 Takara-machi, Kanazawa 920-8640, Japan

^11^ Institute of Human and Social Sciences, Kanazawa University, Kakuma-machi, Kanazawa 921-1192, Japan

^12^ Department of Pediatrics, Kanazawa University, 13-1 Takara-machi, Kanazawa 920-8640, Japan

**Supplementary Notes**

**Supplementary Data 1:**

**Definition of the terms for “sleep variables” in Table 3 & 4 and text.**

Bed time: The time that the toddler went to bed as defined by sleep diary.

Sleep onset time: The time of the start of nighttime sleep.

Wake time: The time of the end of nighttime sleep.

Sleep latency: The duration of time from bedtime to the onset of nighttime sleep.

Nighttime sleep duration: Total hours scored as sleep between sleep onset time and wake time.

Nap duration: The longest sleep period of the day was defined as “nighttime sleep”, and all additional sleep periods of 11 minutes or more were defined as “nap”. Nap duration is sum of all the “naps” of the day.

Total sleep duration: The sum of nighttime sleep duration and nap duration.

Nap onset time: The time of the start of the first nap of the day.

Nap end time: The time of the end of the last nap of the day.

Sleep efficiency: The proportion of actual sleep during bed-in time (%).

WASO: Minutes awake during period between “sleep onset time” and “wake time”.

Night wakings: Number of blocks of contiguous wake epochs lasting more than 5 minutes each.

Daily variation of sleep onset time: Standard deviation of the sleep onset time of each day in the study period.

Daily variation in wake time: Standard deviation of the wake time of each day in the study period

Child activity: Activity counts per minute as recorded by Actigraph at any time of day.

Sleep levels: Sleep or wake status.

Distribution of sleep: Distribution of nap and nighttime sleep over a 24-hour period.

**Supplementary Data 2:**

**Details of the questionnaire used in the present study.**

Brief Toddler Sleep Questionnaire

Please answer the following questions related to your child's sleep over the past one month.

1.How did you feed your child every day?

□ Breastfed □ Formula milk □ Combination of breastfeeding and formula milk □ Fed only solids

2.How did you feed your child before going to bed at night?

□ Breastfed　□ Formula milk □ No feed**ing**

3.Where did your child sleep at night?

□ Child slept **in** own bed in the same room with parents

□ Child had own room, slept in own bed

□ Child slept in separate room from parents, together with siblings in same bed.

□ Child slept in parents’ bed

□ Other style

4.How did your child fall asleep at night?

□ During feeding by breast or with formula milk

□ While being held

□ Wrapped in a towel

□ Alone in own bed

□ With parents in the room

5.What time did your child go to bed?

6.What was your child’s main sleep posture?

□ Supine position　□ Prone position　□ Lateral position

7.How long does it take for your child to fall asleep after going to bed?

（　　）h（　　）min

8.How many times did your child wake up per night?

（ 　）times

9.How long did your child spend awake in total every night?

（　　）h（　　）min

10.What time did your child wake up in the morning?

11.How long did your child sleep at night?

（　　）h（　　）min

12.How many times a day did your child take a nap on average?

13.How much total nap time did your child spend every day?

（　　）h（　　）min

14.Please check all of the following items that apply to your child. ✓.

|  | 1.Usually  (5〜7 times/week) | 2. Sometimes  (2〜4 times/week) | 3. Rarely  (0-1 time/week) | Do you consider this item to be especially problematic for your child? |
| --- | --- | --- | --- | --- |
| Child talked during sleep | □ | □ | □ | yes　　no  not applicable |
| Child cried at night | □ | □ | □ | yes　　no  not applicable |
| Child was restless and moved a lot during sleep | □ | □ | □ | yes　　no  not applicable |
| Child made Jerking movements during sleep | □ | □ | □ | yes　　no  not applicable |
| Child snored loudly | □ | □ | □ | yes　　no  not applicable |
| Child seemed to stop breathing during sleep | □ | □ | □ | yes　　no  not applicable |
| Child snorted and/or gasped during sleep | □ | □ | □ | yes　　no  not applicable |
| After awakening in the middle of the night, child was not able to fall asleep again without being breastfed, or having formula milk, water or food. | □ | □ | □ | yes　　no  not applicable |

15.Do you have concerns about your child’s sleep?

□Yes 　□ A few □ No

**Supplementary Figure 1:**

**Mediation analysis between nap onset time, nap duration, and nighttime sleep duration in term toddlers of approximately 1.5 years of age (*p<0.05, **p<0.01).**


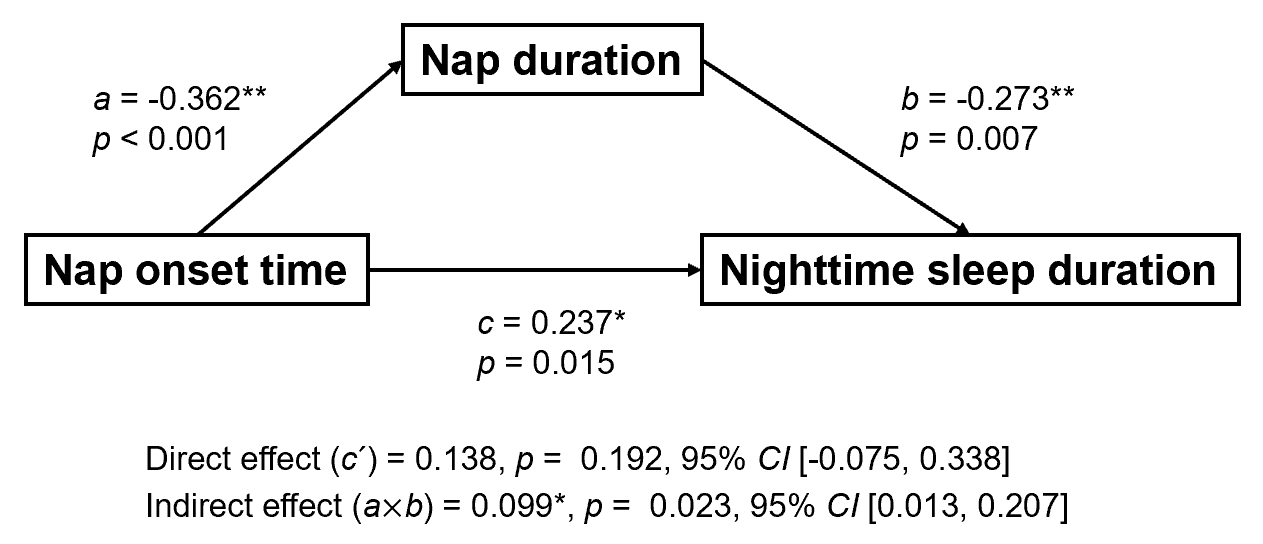


**Supplementary Table 1:**

**The associations of gender/birth profiles, sleep variables, and sleep arrangement factors and total sleep duration evaluated by univariate analysis (**p<0.01, *p<0.05).** Total sleep duration = nap duration + nighttime sleep duration.

| **Sleep-related factors** | **r** | **R^2^** | **p-value** |
| --- | --- | --- | --- |
| **Gender and birth profiles** |  | | |
| **Maternal age at birth** | **-0.152** | **0.023** | **0.120** |
| **Birth order** | **-0.116** | **0.013** | **0.237** |
| **Birth weight** | **-0.045** | **0.002** | **0.646** |
| **Gender** | **-0.022** | **0.000** | **0.826** |
| **Sleep variables** |  | | |
| **Nighttime sleep duration** | **0.847^**^** | **0.718** | **0.000** |
| **Sleep efficiency** | **0.814^**^** | **0.663** | **0.000** |
| **WASO** | **-0.773^**^** | **0.597** | **0.000** |
| **Night wakings** | **-0.650^**^** | **0.423** | **0.000** |
| **Nap duration** | **0.182** | **0.033** | **0.061** |
| **Sleep onset time** | **0.147** | **0.022** | **0.131** |
| **Bed time** | **-0.140** | **0.020** | **0.153** |
| **Wake time** | **0.130** | **0.017** | **0.186** |
| **Daily variation in sleep onset time** | **-0.120** | **0.014** | **0.222** |
| **Nap onset time** | **0.081** | **0.007** | **0.408** |
| **Nap end time** | **0.078** | **0.006** | **0.429** |
| **Daily variation in wake time** | **-0.057** | **0.003** | **0.564** |
| **Sleep latency** | **-0.023** | **0.001** | **0.813** |
| **Sleep arrangement factors** |  | | |
| **Nighttime breast feeding** | **-0.355^**^** | **0.126** | **0.000** |
| **Putting children to sleep with formula** | **0.146** | **0.021** | **0.137** |
| **Child having own room** | **-0.144** | **0.021** | **0.141** |
| **Co-sleeping with parents** | **-0.081** | **0.007** | **0.408** |

**Supplementary Table 2:**

**Logistic regression of total sleep duration of toddlers with gender/birth profiles, sleep variables, and sleep arrangement factors (OR, 95%C.I., **p<0.01, * p<0.05).** Total sleep duration = nap duration + nighttime sleep duration.

| **Variables** | **Model 1,OR (C.I.)** | **Model 2,OR (C.I.)** | **Model 3, OR(C.I.)** |
| --- | --- | --- | --- |
| **Maternal age** | **N.S.** | **N.S.** | **N.S.** |
| **Sleep onset time** |  | **N.S.** | **N.S.** |
| **Nighttime breast feeding** |  |  | **0.296 (0.122, 0.714)**** |
| **Putting children to sleep with formula** |  |  | **N.S.** |
| **Child having own room** |  |  | **0.094 (0.010, 0.850)*** |
| **p-value** | **N.S.** | **N.S.** | **0.003** |
| **R^2^ (Cox-Snell)** | **N.S.** | **N.S.** | **0.106** |
